# An Alternative Medical Diagnosis Method: Biosensors for Virus Detection

**DOI:** 10.3390/bios9020065

**Published:** 2019-05-21

**Authors:** Yeşeren Saylan, Özgecan Erdem, Serhat Ünal, Adil Denizli

**Affiliations:** 1 Department of Chemistry, Hacettepe University, Ankara 06800, Turkey; yeseren@hacettepe.edu.tr; 2 Department of Biology, Hacettepe University, Ankara 06800, Turkey; ozgecanerdem@hacettepe.edu.tr; 3 Department of Infectious Disease and Clinical Microbiology, Hacettepe University, Ankara 06230, Turkey; sunal@hacettepe.edu.tr

**Keywords:** biosensor, medical applications, virus detection

## Abstract

Infectious diseases still pose an omnipresent threat to global and public health, especially in many countries and rural areas of cities. Underlying reasons of such serious maladies can be summarized as the paucity of appropriate analysis methods and subsequent treatment strategies due to the limited access of centralized and equipped health care facilities for diagnosis. Biosensors hold great impact to turn our current analytical methods into diagnostic strategies by restructuring their sensing module for the detection of biomolecules, especially nano-sized objects such as protein biomarkers and viruses. Unquestionably, current sensing platforms require continuous updates to address growing challenges in the diagnosis of viruses as viruses change quickly and spread largely from person-to-person, indicating the urgency of early diagnosis. Some of the challenges can be classified in biological barriers (specificity, low number of targets, and biological matrices) and technological limitations (detection limit, linear dynamic range, stability, and reliability), as well as economical aspects that limit their implementation into resource-scarce settings. In this review, the principle and types of biosensors and their applications in the diagnosis of distinct infectious diseases were comprehensively explained. The deployment of current biosensors into resource-scarce settings is further discussed for virus detection by elaborating the pros and cons of existing methods as a conclusion and future perspective.

## 1. Introduction

Contamination of sources due to viruses is one of the big reasons for diseases which lead to hundreds of thousands of deaths each year. These medical issues have not been solved yet as evidenced by millions of people suffering from several diseases [1]. Nowadays, there is an outstanding rise in the occurrence of infectious diseases which have an important effect on all live species (animals, humans and plants) [2]. Especially in many countries and poor segments of modern society, several contagious diseases such as tuberculosis, malaria, and human immunodeficiency virus are affecting lots of people and also continue to create significant health problems [3]. Viruses are obligate intracellular parasites and need the host cell to propagate and establish genetic material replication. Their complicated protection mechanisms can change very quickly. In response to this situation, viruses are adapted by breaking down and manipulating the host immune reaction. This has led to the emergence of viruses that are adapt at manipulating and subverting host immune responses. In addition, viral infections cause worldwide morbidity and mortality. Notably, some outbreaks have attracted attention in the last years; influenza A H1N1 subtype in 2009 and Ebola virus outbreak in 2014 [4].

The early determination of pathogenic agents like bacteria and viruses is crucial for clinical point-of-care purposes [5]. Polymerase chain reaction [6], enzyme-linked immunosorbent assay [7], reverse transcription polymerase chain reaction [8], and different biosensor technologies are used to detect or remove viruses [9,10,11,12,13]. Antigen–antibody or receptor–ligand-based virus detection tools can be found on the market. Regardless of their numerous favorable circumstances, biomolecules possess essential defects in terms of utilization and stability [14]. Because of the requirement for fast diagnosis and developments in more selective, stabile and economical biosensor technology, new recognition elements have been studied to improve recognition in biosensing [15]. Biosensors are analytical devices which consist of an analyte, bioreceptor, transducer, and measurable signal [16]. The analyte is captured and biological responses are converted into signals. Regarding the transduction principles, biosensors can be classified into three main class as optic [17,18,19], electrochemical [20,21,22], and piezoelectric [23,24,25,26]. Over the previous two decades, biosensors have turned out to be important to detect distinctive analytes, for example, explosives [27,28], proteins [29,30], nucleic acids [31,32], cancer biomarkers [33,34], bacteria [35,36], viruses [37,38], and toxins [39,40] in food processing [41], environmental monitoring [42], clinical diagnostics [43,44] and the fight against bioterrorism [45].

In this review, the principle and types of biosensors are explained first and then the new biosensor types and technologies are extensively discussed according to the latest research findings for rapid assessment of the medical applications to diagnosis infectious diseases. Finally, conclusion and future perspectives are mentioned to compare commercial biosensors and sum up the review. 

## 2. Principle of Biosensor

A biosensor is an analytical device with three major modules: (i) a sensing bioreceptor; (ii) a transducer; and (iii) a detector with a digital output. Principally, target analyte interacts with bioreceptor [46], and the detecting component part specifically recognizes the analyte through a reaction, specific adsorption, or another process such as physical/chemical interaction. Then, the transducer translates molecular changes to a quantifiable signal measured by the digital detector module [47]. The subject of the transduction principles can be separated as electrochemical, piezoelectric, optical, thermal, micromechanical, and magnetic. Biosensors provide multiple capabilities, including exceptional performance, user-friendly operation, rapid response, high sensitivity and specificity, portability, relatively compact size, and real-time analysis [48]. Nowadays, researchers mean to enhance the sensitivity and specificity of the techniques by focusing on the biosensor development and fabrication quality, expanding the affinity between creating innovative surface chemistries, and using nanomaterials such as nanofilm [49], nanoparticle [50] or quantum dot [51] for signal amplification.

### 2.1. Types of Biosensors

#### 2.1.1. Electrochemical Biosensors

Electrochemical biosensors have been utilized for a long time to reach a wide range of applications in various areas. These biosensors represent a typical platform for the construction of biosensors, which include semi-conductors and screen-printed electrodes [52]. Briefly, these biosensors monitor any alterations in dielectric properties, dimension, shape, and charge distribution while the antibody–antigen complex is formed on the electrode surface. They can be classified into four major groups including potentiometric, amperometric, cyclic voltammetry, and impedimetric transducers [53]. These biosensors have been employed to detect a variety of biological targets, including proteins, cancer biomarker, nucleic acid, and so on [54,55,56]. 

#### 2.1.2. Piezoelectric Biosensors 

One of the most common piezoelectric biosensors is the quartz crystal microbalance biosensor, which measures any mass change and viscoelasticity of materials by recording frequency and damping change of a quartz crystal resonator [57]. Due to high sensitivity to environmental conditions, the sensing mechanism significantly requires isolation equipment that minimizes any hindrance factors such as vibration. These biosensors have been used in a wide variety of applications to detect targets including hormone, bacteria, cell, and so on [58,59,60]. 

#### 2.1.3. Optical Biosensors

Optical biosensors focus on the measurement of a change in the optical characteristics of the transducer surface when the analyte and recognition element form a complex. These biosensors can be divided into two groups. For example, a signal generation depends on the formation of a complex on the transducer surface in the direct optical biosensor. The indirect optical biosensors are mostly designed with various labels such as fluorophores or chromophores to detect the binding events and amplify the signal. Although indirect biosensing methods can produce higher signal levels, they suffer from non-specific binding and high reagent cost of labelling step [61]. In the literature and the market, there are multiple optical biosensors, including optrode-based fiber optical biosensors, evanescent wave fiber optical biosensors, time-resolved fluorescence, the resonant mirror optical biosensor, interferometric biosensors and surface plasmon resonance biosensors. Their detection window is so versatile, and they sense multiple types of biomolecules from physiological and biological specimens [62].

### 2.2. Importance of Biosensors in the Medical Diagnosis

Biosensor innovation developed many years ago. Several researchers including biologists, chemists, physicists, medical doctors have been joined to use the biosensor as an original application in different fields such as doping analysis [63], diagnosis [64], food safety [65], laboratory medicine [66], and so on. Among them, certainly, clinical examinations have additionally been explored as a powerful application area. Because of the requirement for fast analysis and enhancements in detecting characteristics, i.e., stability, selectivity, and being profitable, new recognition elements and arrangements of them have been studied to enhancement recognition in biosensor systems. The innovation of new recognition components and the utilization of nanotechnology have contributed to the improvement in biosensors. The analytical performance of biosensors has increased in detecting characteristics with this combination. These highlights make biosensors appropriate for point-of-care diagnostics because they can achieve quick and multi-analyte detection [67]. There are different types of biosensors including optical [68,69], electrochemical [70,71], piezoelectric [72,73], magnetic [74,75], micromechanical [76,77], and thermal [78,79,80,81] for medical diagnosis.

## 3. Latest Applications of Biosensors on Virus Detection

### 3.1. Human Immunodeficiency Virus

**Figure 1:**
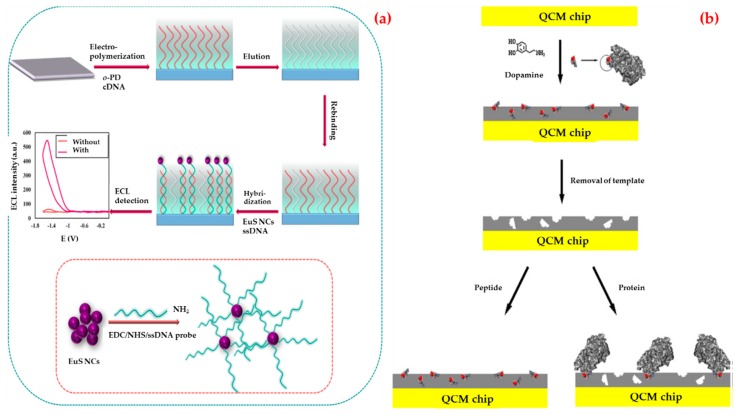
Schematic representations of the electro-chemiluminescence (**a**) and quartz crystal microbalance; (**b**) biosensors for the detection of HIV-1. Republished with permission from [84,85]; permission conveyed through Copyright Clearance Center, Inc.

Human immunodeficiency virus (HIV) is a member of a subset of retroviruses named lentiviruses. Lentiviruses also mean a slow virus; and it indicates the period between the start of the infection and the emergence of the symptoms. HIV infects the CD4^+^ T cells and starts to replicate quickly behind entering the bloodstream [82]. The final stage of the infection acquired immunodeficiency syndrome (AIDS) is one of the outstanding public health problems. According to the World Health Organization, more than 35 million people have been infected by HIV up to now. In 2017, 940,000 people died from HIV-related causes globally [83]. There is an urgent need for a more sensitive and specific biosensing platform to diagnose HIV. There are two types of HIV viruses and HIV-1 is the most common type to cause disease. There are several recent research studies about this virus detection using biosensors. For example; Babamiri et al. developed an imprinted-based electro-chemiluminescence biosensor for HIV-1 gene detection [84]. They used HIV aptamer as a template and o-phenylenediamine as a functional monomer (Figure 1a). After the experiments, they observed that the response significantly increased after the hybridization reaction. They achieved a very sensitive HIV gene detection (0.3 fM) in a range of 3.0 fM to 0.3 nM. The prepared biosensor showed good specificity for HIV detection when compared with the non-complementary sequences. They also tested serum samples and obtained high recoveries in the range of 95–101.2%. 

Glycoprotein41 (Gp41) is the transmembrane protein of HIV-1 and have a significant role in membrane fusion between infected cells and virus. The extent of AIDS progression and the efficacy of therapeutic intervention can be monitored with Gp41. Lu et al. developed a biosensor with the purpose of detecting HIV-1-related Gp41 [85]. They modified a quartz crystal microbalance biosensor surface with a synthetic peptide which is analogous to 579–613 residues of Gp41 by epitope imprinting method (Figure 1b). According to the results, they showed that an imprinted film has a great affinity to the target peptide and can bind Gp41 protein selectively. They also found that the limit of detection as 2 ng/mL. 

An optical sensing platform was demonstrated to detect HIV-1 from biological samples by Shafiee et al. [86]. In this study, they observed that when the intact virus was adsorbed by the surface, this situation induced a shift in the resonant peak wavelength value. This value can be detected with 10 pm wavelength resolution. Both biomolecular layers and even low concentrations of viruses can be detected with this biosensor. They also examined HIV-1 detection in serum and phosphate buffered saline samples with viral loads ranging from 10^4^ to 10^8^ copies/mL. Table 1 provides a comparison of biosensor platforms for human immunodeficiency virus detection.

### 3.2. Hepatitis

It has been known since at least the 1940s that blood and plasma samples may contain a virus that causes transient and chronic hepatitis, also known as post-transfusion hepatitis [87]. Hepatitis B is one of the major infections of humanity, assessed to cause around 800,000 deaths for every year for the most part from liver malignant growth and cirrhosis. Nearly 15–40% of infected patients will develop liver failure, liver cirrhosis, or hepatocellular carcinoma and 15–25% will eventually die [88].

Hassen et al. reported a method based on DNA hybridization in order to detect the hepatitis B virus using non-faradic electrochemical impedance spectroscopy [89]. They first modified DNA probes with biotin on streptavidin-based magnetic nanoparticles and then immobilized nanoparticles onto the bare gold electrode using a magnet. After the characterization experiments, they showed that the successful immobilization of DNA probes and the hybridization with different concentrations of complementary DNA. Furthermore, they demonstrated that non-faradic impedance spectroscopy can be detected 50 picomoles of HBV DNA on a sample of 20 μL and saturation was reached 12.65 nmole/mL for the same quantity of immobilized DNA probes. 

In addition, Tam et al. investigated the detection of hepatitis B surface antigen antibody ability using a surface plasmon resonance biosensor [90]. They obtained a linear performance in a range of 0.00098–0.25 mg/L, and a seven-fold higher limit of detection value, that a two-fold increase in the coefficient of variance of the replicated results, as compared with enzyme-linked immunosorbent assay. In addition, they reported that the evaluation of the assay for specificity had no cross-reactivity with other antibody tested. 

**Figure 2:**
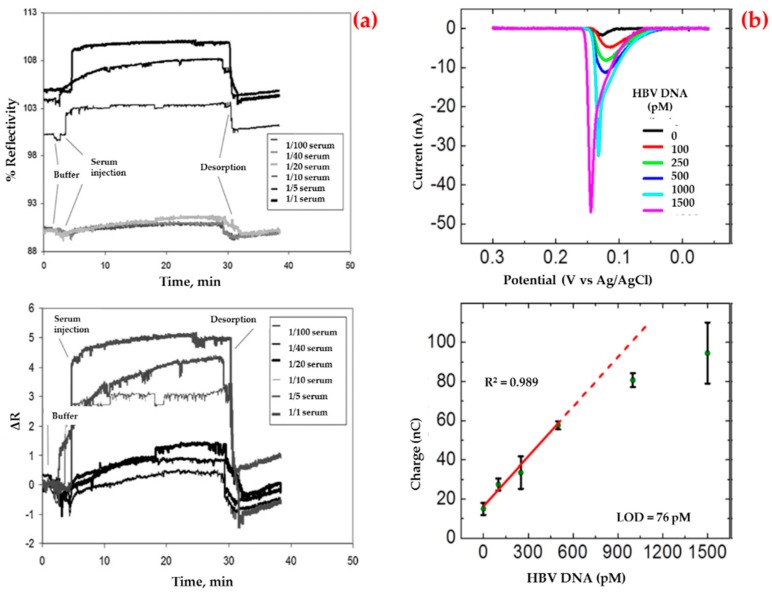
Sensorgrams for the interaction between hepatitis B surface antibody positive human serum and biosensor (**a**) and voltamograms for the hepatitis B virus DNA sandwich assay in a conventional electrochemical biosensor (**b**). Republished with permission from [91,92].

Uzun et al. also detected a hepatitis B surface antibody employing a surface plasmon resonance biosensor for the diagnosis of hepatitis in human serum [91]. They performed kinetic studies using the hepatitis B surface antibody positive human serum samples (Figure 2a). According to the mathematical calculations, they showed that this biosensor had some surface homogeneity, obeyed the Langmuir adsorption isotherm model and had a low detection limit value (208.2 milli-international unit per milliliter, mIU/mL). Finally, they performed control experiments using non-immunized (hepatitis B surface antibody negative) serum sample and the results showed that the biosensor did not give any noticeable response to negative serum. 

On the other hand, Li et al. developed an impedimetric biosensor that was modified with gold nanoparticles for sequence-selective DNA hybridization related to the hepatitis B virus [92]. They presented that the biosensor had a high correlation coefficient in a low concentration range (Figure 2b), repeatable responses and provided a suitable surface for more DNA binding. In addition, the selectivity of this biosensor was investigated in the presence of the target and the other DNA sequences.

Istek et al. prepared a paper-based electrochemical biosensor to detect DNA from the hepatitis B virus [93]. They reported that this biosensor had four necessary attributes. First of all, they combined design with paper folding for timing incubation. In the second part, two stages of amplification were done; silver nanoparticle labeled was provided a maximum amplification factor and magnetic microbeads were captured the probes. Third, the fact that any enzyme or antibody was not used in the study improved the stability, speed, and robustness of the biosensor. Lastly, just a single sample incubation step was necessary before detection was started. They found the detection limit value as 85 pM. 

Zengin et al. reported a biosensor for DNA sequence of the hepatitis B virus detection that depended on a sandwich assay and surface-enhanced Raman scattering. Firstly, they prepared a temperature-responsive hybrid silicon substrate to immobilize DNA strand at the gold nanoparticle surface. Then, the sandwich strategy was carried out for the detection of target DNA with high surface-enhanced Raman scattering signals. They measured the lowest hepatitis B virus DNA concentrations at fM levels at different temperatures. They claimed that this highly sensitive and robust platform can be extended to detect other biomolecules and chemical species without any labeling [94].

Liu et al. developed a thermosensitive surface-imprinted polymer-based biosensor for hepatitis A virus monitoring. They regulated the recognition performance of the biosensor by temperature control for virus capture. In addition, they calculated a very low (1.1 pM) detection limit value. They also successfully used this biosensor to detect the additional hepatitis A virus from a dilution of human serum and obtained high recoveries in the range of 90.8 to 108.3% at three levels of viruses. Finally, they addressed the problems of high non-specific adsorption and long duration of detection process [95]. To sum up and compare these studies, Table 2 provides detailed information about biosensor platforms for hepatitis virus detection.

### 3.3. Ebola

Ebola infections cause severe illness in humans. Patients have general influenza-like side effects before a fast progression of disease that is characterized by multiple organ failure, haemorrhage, and a shock-like syndrome after an incubation time (3–21 days) [96]. The largest outbreak of Ebola virus infection has 15,935 reported cases and 5689 deaths in 2014 [97]. Such innovative advantages would be ideal for fast, point-of-care identification and determination of the Ebola virus, specifically in environments with insignificant infrastructure, for example, in general well-being or emergency response circumstances [98]. 

**Figure 3:**
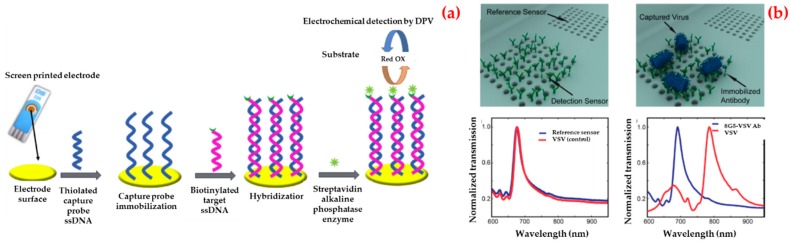
Schematic representation of different steps for the fabrication of electrochemical biosensor (**a**) and three-dimensional renderings and the experimental measurements illustrate the detection scheme using optofluidic nanoplasmonic biosensors based on resonance transmissions due to extraordinary light transmission effect (**b**). Republished with permission from [99,100].

For instance, Ilkhani et al. fabricated an electrochemical biosensor for Ebola virus DNA diagnostics by an enzyme-amplified detection [99]. As depicted in Figure 3a, they labeled the biotinylated hybrid with a streptavidin–alkaline phosphatase conjugate. They optimized all the experiment steps using electrochemical impedance spectroscopy and then obtained a low detection limit value (4.7 nM) using this biosensor and the standard deviation of the blank solution. They finally performed selectivity and reproducibility of the electrochemical biosensor. 

Additionally, Yanık et al. demonstrated an optofluidic biosensor platform which directly detected whole viruses from biological media [100]. The detection can be done at clinically related concentrations and any sample preparation is needed. The prepared biosensor depended on a light transmission impact in plasmonic nanoholes and used group-specific antibodies. They enveloped the detection of small RNA viruses (vesicular stomatitis virus and pseudo typed Ebola) within a dynamic range spanning three orders of magnitude. For these experiments, they immobilized antibodies against the Ebola glycoprotein on the biosensors and transmission spectra were collected after the washing process (Figure 3b). 

Cai et al. also reported a study about amplification-free detection and quantification of Ebola virus on clinical samples [101]. A microfluidic chip was used for sample preparation and the pre-concentration of the virus. Behind this step, single nucleic acid fluorescence detection in liquid-core optical waveguides on a silicon chip was done within ten minutes. They also showed that this biosensor had an outstanding specificity and a low limit of detection value (0.2 pfu/mL). There is a comparison of biosensor platforms in Table 3 for Ebola virus detection.

### 3.4. Zika

Zika virus is a mosquito-borne virus that was first identified in Uganda. Prior to 2007, just sporadic human sickness cases were accounted for from nations in Africa and Asia. The first documented outbreak of Zika virus infection was reported in the Federated States of Micronesia in 2007 [102]. Emerging infectious illnesses, including the ongoing West African Ebola virus outbreak, and now, the Zika virus epidemic spanning the Western Hemisphere, has brought renewed regarding the need to create simplified diagnostic tests for use in low-resource settings [103].

**Figure 4:**
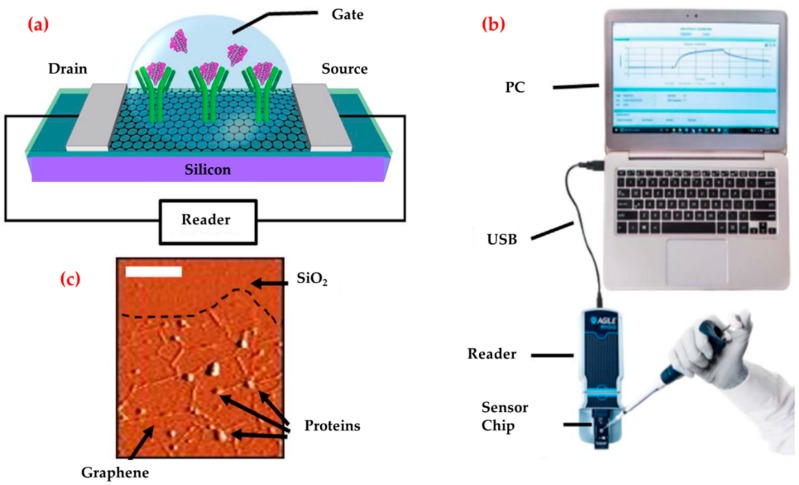
Diagram of the graphene biosensor (**a**), the illustration of the entire biosensor system (**b**) and an atomic force microscope image of the graphene after protein attachment (**c**). Republished with permission from [104].

A profitable and portable graphene-enabled biosensor was developed by Afsahi et al. to detect the Zika virus with a highly specific immobilized monoclonal antibody [104]. They covalently linked monoclonal antibodies to graphene for native Zika viral antigens detection (Figure 4). They measured low antigens concentrations (450 pM). They also demonstrated potential diagnostic applications by measuring Zika antigen in a recreated human serum and the clinical performance of the biosensor by measuring Zika antigen in human serum and also validated the selectivity with Japanese Encephalitis NS1.

Further, Kaushik et al. presented an electrochemical biosensor for Zika virus protein detection [105]. They performed electrochemical impedance spectroscopy to measure the electrical response of the biosensor as a function of protein concentrations and showed that this biosensor detected Zika virus protein selectively in a detection range of 10 pM to 1 nM and a detection limit value of this biosensor lower than 10 pM with high sensitivity. 

Song et al. also reported a reverse-transcription loop-mediated, isothermal amplification (RT-LAMP) assay for diagnosis of Zika virus as a disposable cassette [106]. A chemically heated cup was used for thermal control of the cassette. Because of this, no electrical power was needed. The researchers designed a set of new RT-LAMP primers for Zika virus, the envelope protein-coding region. They demonstrated the utility of this diagnostic system by detecting the virus in oral samples with a sensitivity of 5 pfu in less than 40 min. Table 4 compares biosensor platforms for Zika virus detection.

### 3.5. Norovirus

Norovirus, is a human enteric pathogen that causes significant disease across health care. Norovirus is an important reason for morbidity because of acute gastroenteritis both inside health care institutions and in the more extensive network [107]. Norovirus contamination happens generally from contaminated water or food, and infection can easily spread from person to person through fecal or oral ways [108]. The most important reason for acute gastroenteritis outbreaks and sporadic disease was identified as Noroviruses worldwide [109]. Numerous examinations have concentrated on giving promising alternative techniques to detect noroviruses in more sensitive and accurate ways like aptamer-based electrochemical biosensors and microfluidic chips. 

**Figure 5:**
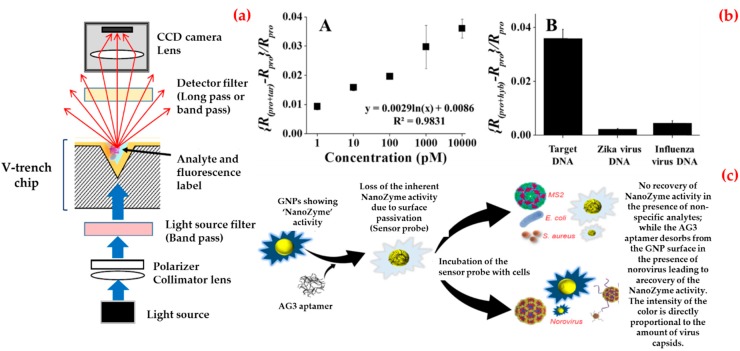
Schematic diagram of a V-trench biosensor (**a**); norovirus DNA detection: sensitivity and specificity tests (**b**); and working principle of the biosensor for norovirus detection (**c**). Republished with permission from [110,111,112].

Exemplarily, Ashiba et al. investigated a surface plasmon resonance biosensor to detect norovirus virus-like particles [110]. They designed this biosensor that used a chip equipped with a V-shaped trench (Figure 5a). They selected an excitation wavelength as 390 nm to excite surface plasmon resonance on an aluminum film of the biosensor. They calculated the minimum detectable concentration as 0.01 ng/mL, which corresponds to 100 virus-like particles included in the detection region of the V-trench. 

Besides, Lee et al. synthesized binary-nanoparticle-decorated carbon nanotubes and applied as a biosensing platform [111]. They first aligned on a platinum-interdigitated electrode with gold/magnetic nanoparticles-carbon nanotubes, and then, attached a thiol-group-functionalized probe DNA to the gold nanoparticle surface to obtain this hybrid structure. As shown in Figure 5b, they monitored different concentrations of target DNA (1 pM–10 nM), and calculated the limit of detection value as around 8.8 pM. They also confirmed specificity using other mismatched DNA sequences. 

In addition, Weerathunge et al. proposed a recent colorimetric biosensor platform for rapid and ultrasensitive detection of the infective murine norovirus [112]. They combined the enzyme-mimic catalytic activity of gold nanoparticles with high target specificity of a murine norovirus aptamer to create biosensor and observed that this biosensor produced a blue color in the presence of norovirus (Figure 5c). They also calculated the limit of detection value as three viruses per assay that equal to 30 viruses/mL of sample and experimentally-demonstrated limit of detection value as 20 viruses per assay equal to 200 viruses/mL. Furthermore, they showed the robustness of the norovirus biosensor by testing its performance in the presence of other microorganisms in human serum and shellfish homogenate. Table 5 contributes a comparison of biosensor platforms for Norovirus detection.

### 3.6. Influenza

Influenza is a viral infectious disease considered as a wellspring of numerous medical issues and tremendous financial burden [113]. Regular techniques are deficient for in-field detection of the infections and generally suffer from being difficult and time-consuming. Accordingly, researches pointing to improve effective alternatives to conventional techniques are instantly necessary [114]. 

**Figure 6:**
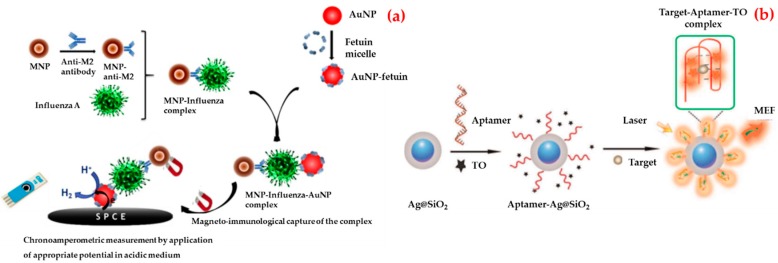
Schematic illustration of the strategy of the gold nanoparticle-based chronoamperometric biosensor development for influenza virus (**a**) and the preparation of aptamer-based biosensor and the determination of rHA protein of H5N1 (**b**). Republished with permission from [115,117].

For example, Sayhi et al. developed a method with the aim of isolation and detection of influenza A virus H9N2 subtype [115]. They first attached an anti-matrix protein 2 antibodies to iron magnetic nanoparticles and used them in order to isolate the influenza virus from an allantoic fluid. Afterwards, Fetuin A was attached to an electrochemically detectable label, gold nanoparticles, to detect the virus tacking advantage from fetuin-hemagglutinin interaction (Figure 6a). They isolated complex and treated it with an acid solution to collect gold nanoparticles for deposition onto a screen printed carbon electrode. They reported that this biosensor allows the rapid detection of influenza virus A/H9N2 at a less than 16 Hemagglutinin Units (HAU) titer. 

Also, Tam et al. described a study about DNA immobilization using carbon multi-walled nanotubes for influenza virus detection [116]. They attached the DNA probe onto the biosensor and characterized the interaction by Fourier transform infrared and Raman spectrometry analyses. They detected the hybridization of the DNA probe and the target DNA by changes in the conductance on the surface of biosensors leading to the change in the output signal of the system and calculated the detection limit value as 0.5 nM. 

Pang et al. designed a fluorescent biosensor for the detection of recombinant hemagglutinin protein of the H5N1 influenza virus in human serum [117]. As shown in Figure 6b, they followed several steps to prepare a fluorescent biosensor. Firstly, they immobilized guanine-richen anti-recombinant hemagglutinin aptamers by SELEX on the surface of the silver–silicon dioxide nanoparticles. Then, they used thiazole orange as a fluorescent tag. They observed that thiazole orange was free with no fluorescence emission in the absence of recombinant hemagglutinin protein and the aptamer strand bound recombinant hemagglutinin protein formed a stable G-quadruplex complex when recombinant hemagglutinin protein was added to the solution. They operated the detection of recombinant hemagglutinin protein of the H5N1 influenza virus in both in aqueous buffer and human serum with the detection limit value of 2 and 3.5 ng/mL.

Furthermore, Vollmer et al. reported an optical detection method of Influenza A virus [118]. They observed that the binding of single virions from discrete changes in the resonance frequency of a whispering gallery mode excited in a microspherical cavity. They also found that the magnitude of the discrete wavelength-shift signal can be sufficiently enhanced by reducing the microsphere size. They confirmed that a reactive sensing mechanism with inverse dependence on mode volume with virus-sized polystyrene nanoparticles. 

Bai et al. prepared a portable surface plasmon resonance biosensor by using an aptamer for avian influenza virus H5N1 detection in poultry swab samples [119]. They fabricated the biosensor using selected aptamers that were biotinylated and then immobilized on the biosensor gold surface coated with streptavidin. The immobilized aptamers captured AIV H5N1 in a sample solution, which caused an increase in the refraction index. After optimizing the streptavidin and aptamer parameters, the results showed that the refraction index value was linearly related to the concentration of AIV in the range of 0.128–1.28 hemagglutinin unit (HAU). Table 6 provides a comparison of biosensor platforms for Influenza virus detection.

### 3.7. Dengue

Dengue fever has re-emerged as a major public health challenge worldwide; with 2.5 billion people at risk of infection, more than 100 million cases and 25,000 deaths were reported annually [120]. Dengue virus is a member of the Flavivirus genus of single-stranded positive-sense RNA viruses that cause visceral and central nervous system disease in humans. Dengue virus infections result in either inapparent disease (up to 75% of infections) or a spectrum of clinical illnesses ranging from self-limited dengue fever to severe dengue, a potentially lethal hemorrhagic and capillary leak syndrome previously termed dengue hemorrhagic fever and dengue shock syndrome [121]. Detection of IgM and dengue NS1 glycoprotein based on rapid diagnostic tests and ELISA methods are the most widely used dengue assays in many countries. Because of this, many researchers have delved into biosensors as an alternative new technology for the detection of Dengue virus and dengue antibodies since this technique has several advantages such as higher sensitivity, cost-effective, simple fabrication, possible miniaturization, a rapid outcome with quantitative analysis and possible on-site monitoring [122].

**Figure 7:**
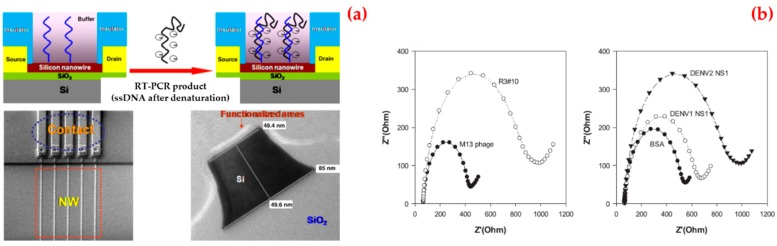
A schematic diagram of the sensor, scanning electron microscope image of nanowire arrays and the corresponding contact lines, transmission electron microscope image of nanowire after surface functionalization (**a**); selectivity test of selected phage clones: phage particles were incubated with dengue virus NS1 proteins, dengue virus type 1 NS1 and type 2 NS1 protein (**b**). Republished with permission from [123,124,126].

For instance, Zhang et al. presented a silicon nanowire biosensor for the detection of dengue serotype 2 [123]. They first covalently attached a peptide nucleic acid onto the silicon nanowire surface. After that a complementary fragment of dengue serotype 2 was obtained and applied to the peptide nucleic acid-functionalized silicon nanowire. They verified the hybridization by measuring the resistance change of the silicon nanowire biosensor before and after the binding of the dengue serotype 2 to the peptide nucleic acid sequence (Figure 7a). They reported that the silicon nanowire biosensor can detect below 10 fM concentrations of the amplicons. 

Lim et al. used polyvalent phage to identify the affinity of peptides to NS1 protein [124]. They showed that the peptide was rich in basic residues from amino acid sequence analysis. Among all the peptides tested, the selected phage showed the greatest decrease in current in cyclic voltammetry and an increase in impedance in electrochemical impedance spectroscopy upon binding to NS1 proteins. They also revealed that phage clones were more specific towards NS1 proteins, as compared to bovine serum albumin or the M13 wild type (Figure 7b). 

In addition, Deng et al. developed an anodic aluminum oxide membrane sensing platform for DNA detection [125]. They coated the alumina membrane with platinum electrodes to eliminate the solution resistance outside the nanopores and then used the electrochemical impedance technique to monitor the impedance changes within the nanopores upon DNA binding. They demonstrated that the pore resistance linearly increases in response to the increasing concentration of the target DNA. Furthermore, the biosensor selectively differentiates the complementary sequence from single base mismatched strands and non-complementary strands. 

Jahanshahi et al. proposed a surface plasmon resonance biosensor for the detection of the anti-dengue virus in human serum samples [126]. They used four dengue virus serotypes as ligands on a biosensor. According to the results, they showed that a minimized serum volume serum from a dengue patient is required to indicate surface plasmon resonance biosensor angle variation to determine the ratio of each dengue serotype in samples with high sensitivity (83–93%) and specificity (100%). Table 7 also supplies a comparison of biosensor platforms for Dengue virus detection.

### 3.8. Other Viruses

**Figure 8:**
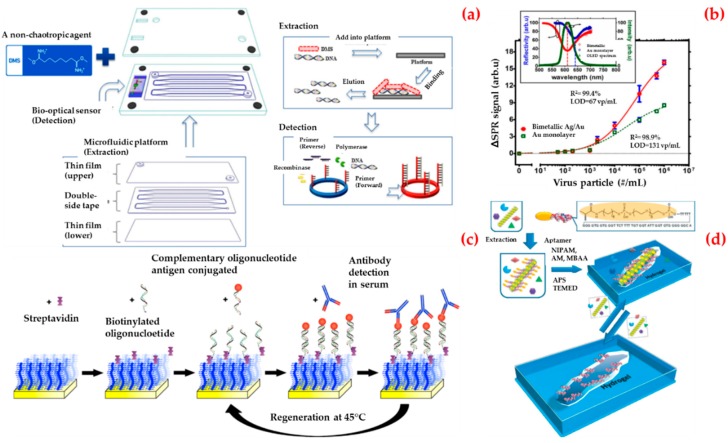
Schematic representation of the sensing platform for sample processing and detection, viral DNA extracted from the platform and followed by an optical biosensor for the detection of human adenovirus in a single cartridge (**a**); curve fitting of the series concentrations of the human enterovirus 71 samples (**b**); the preparation of the biosensor for detection of the Epstein–Barr virus infection (**c**); the imprinting process for creation virus responsive hydrogels (**d**). Republished with permission from [130,131,132,133].

The detection of viruses is of interest for a number of fields including biomedicine, environmental science, and biosecurity [127]. In addition to expensive equipment and expert personnel, systems where the results can be read with the naked eye are of particular interest [128,129]. Jin et al. reported a virus diagnostic system based on an optical biosensor and microfluidic sample processing for human adenovirus detection (Figure 8a) [130]. They first obtained viral DNA from human adenovirus samples using a different extraction technique and then they observed that the optical biosensor can detect ten copies of human adenovirus in clinical samples in half an hour. Finally, they validated the clinical utility of the virus diagnostic system in thirteen human samples (ten of them with human adenovirus and three of them with another pathogen). They claimed that the virus diagnostic system offers a rapid and sensitive diagnostic platform for viral DNA analysis with low cost, simplicity, short assay time, and without the need for complex instruments. 

Prabowo et al. presented a label-free detection and rapid quantification method for human enterovirus 71 using a portable surface plasmon resonance system [131]. They selected the major capsid protein of human enterovirus 71 as a biomarker. They reduced the experimental time required for the human enterovirus 71 quantification from six days to several minutes. As depicted in Figure 8b, they also established a detection limit value of approximately 67 virus particles per milliliter (vp/mL) and, finally, they obtained a detection limit value of major capsid protein of human enterovirus detection in the surface plasmon resonance biosensor as 4.8 pg/mL.

Riedel et al. introduced a biosensor platform based on the surface plasmon resonance system for the diagnosis of different stages of Epstein–Barr virus infections in clinical serum samples [132]. This was achieved by simultaneous detection of the antibodies against three different antigens present in the virus and then the biosensor was attached via hybridization of complementary oligonucleotides (Figure 8c). By this way, they used the same sensing surface repeatedly. They claimed that this approach will serve as a prototype strategy for the development of biosensors for medical applications. 

Bai et al. developed a double imprinting method based on a virus-imprinted hydrogel into a sensor by using imprint-lithography techniques (Figure 8d) [133]. They used a simple laser transmission apparatus to measure diffraction and read by the naked eye to detect the apple stem pitting virus at concentrations as low as (10 ng/mL). They reported that the limit of the detection value of this system was lower than other commercial antigen-binding methods. 

İnan et al. prepared a microfluidic filter device to detect and quantify human papilloma virus 16 E7 antibodies from whole blood as a non-invasive assisting technology for diagnosis of human papilloma virus-associated malignancies [134]. They detected human papilloma virus 16 E7 antibody down to 2.87 ng/mL. They also validated their platform in clinical patient samples and provided significantly high responses as compared to control samples. They reported that this platform can be potentially implemented as a pretesting tool to identify high-risk groups for broad monitoring of human papilloma virus-associated cancers in resource-constrained settings. 

Birnbauer et al. reported a study about the preparation of micro total analysis biosensor system to continuously monitor viral contamination. They combined microfluidics containing integrated native and imprinted polymer with contact-less dielectric biosensors for human Rhinovirus serotype 2 detection. They showed that viral and dissociation binding can be readily detected for specific frequencies. They completed the removal of the virus to demonstrate the reusability of the biosensor following a fifty-fold increase [135].

Feng et al. studied fluorescent detection of Japanese encephalitis virus by surface molecularly imprinted polymer-based biosensor. They first modified silica microspheres with dansyl chloride and then polymerized using available monomer and cross-linker mixture with Japanese encephalitis virus in mild condition. After the polymerization, they performed detection experiments and showed that this biosensor can selectively recognize Japanese encephalitis virus in the presence of other competitive agents (Hepatitis A virus, Simian virus 40 and Rabies virus) and sensitively detect with a low (pM) detection limit level [136]. Finally, Table 8 shows a comparison of biosensor platforms for other virus detections.

## 4. Conclusions

In medical diagnosis, testing for specific biomarkers is performed in centralized laboratories using large automated clinical analyzers that are generally based on DNA or protein microarrays including traditional immunoassay methods (radioimmunoassay or enzyme-linked immunoassay) which need to use labels. They usually allow multiplex detection of several analytes but require trained staff, long time, and a lot of effort. On the other hand, rapid development of nanotechnology and biotechnology has clearly improved the design and fabrication of new devices for biosensing in medical applications. Biosensors are desirable platforms which have several advantages including high detection capability, stability, simplicity, reliability, and affordability, and they can be designed without negatively affecting the sensitivity and the reproducibility of standards in clinical analysis. Biosensor feasibility seems to start leaving the proof-of-concept stage and a growing number of analytes have already been detected several biomolecules including proteins, hormones, and nucleic acids, and also more complex molecules such as exosomes, bacteria, viruses, or cells which demonstrate the versatility of the biotechnology. Furthermore, there are some examples for portable biosensors to determine some results (blood glucose levels or blood coagulation) for patient self-testing. Though many different biosensor platforms have been developed in this concept, a clear leader has not yet been established in clinical routine practice but medical diagnostics show an enormous research field that still has to face many unmet challenges necessary for the development and commercialization of devices.

In this review, the recent developments of biosensors were extensively overviewed for different virus detection in medical applications. Compared with conventional techniques, these biosensor platforms show more promising applications for improving human health for many countries and rural areas of cities.

## 5. Future Perspectives

A powerful biosensor should have user-friendly properties together with high performance components. These important features have been adapted for various specific applications such as infection-related medical diagnosis. In addition, a suitable biosensor platform for microbial diseases is difficult to find in the medical market due to the investigation of bacteria or virus-related marker profiles. Until these profiles are established, biosensor platforms should be kept adaptable. Tests for biomarkers are usually carried out in laboratories with automated analyzers. Most of them are based on microarray or immunoassay methods. Beside the disadvantages of these methods, a portable platform provides correct results within a short time. We needed to wait a long time for finding out the cause of some infectious diseases. There are still some challenges to overcome, and some portable biosensor designs have recently appeared at the research level. However, the progression is rather slow, a significant advancement in smart phone technology as mobile health diagnostics, in particular, for deployment at developing countries and low-resource decentralized settings. The exponential growth in the development of mobile applications and the affordability of these platforms are called to revolutionize health delivery and open the door to a new stage in global health access.

## Figures and Tables

**Table 1:** Comparison of biosensor platforms for HIV detection.

Biosensor Type	Virus/Sample	Recognition Element	Dynamic Range	Detection Limit	Portability	Reference
Electrochemical	HIV-1	HIV aptamer	3.0 fM–0.3 nM	0.3 fM	No	[84]
Piezoelectric	HIV-1	Glycoprotein 41	2–200 ng/mL	2 ng/mL	No	[85]
Optical	HIV-1	Glycoprotein 120 antibody	10^4^–10^8^ copies/mL	10^5^ copies/mL	Yes	[86]

**Table 2:** Comparison of biosensor platforms for hepatitis virus detection.

Biosensor Type	Virus/Sample	Recognition Element	Dynamic Range	Detection Limit	Portability	Reference
Electrochemical	Hepatitis B DNA	Streptavidin	2.53–50.6 nmol/mL	50 pmol	No	[89]
Optical	Hepatitis B surface antigen	*Pichia pastoris*-derived hepatitis B surface antigen	0.00098–0.25 mg/L	0.00781 mg/L	No	[90]
Optical	Hepatitis B antibody	Hepatitis B surface antibody	0–120 mIU/mL	208.2 mIU/mL	No	[91]
Electrochemical	Hepatitis B DNA	Silver nanoparticles	0–1.5 nM	85 pM	Yes	[92]
Electrochemical	Hepatitis B DNA	Gold nanoparticles	1–20 μg/mL	85 pM	No	[93]
Optical	Hepatitis B DNA	Capture DNA strand	0.001 fM–6.0 μM	0.44 fM	No	[94]
Optical	Hepatitis A	Thermosensitive surface imprinted polymer	5–25 pM	1.1 pM	No	[95]

**Table 3:** Comparison of biosensor platforms for Ebola virus detection.

Biosensor Type	Virus/Sample	Recognition Element	Dynamic Range	Detection Limit	Portability	Reference
Electrochemical	Ebola (DNA)	Biotinylated target strand DNA	0–5 nM	4.7 nM	No	[99]
Optical	Ebola	Immobilized anti-viral immunoglobulins	10^6^–10^9^ pfu/mL	10^5^ pfu/mL	No	[100]
Optical	Ebola	Fluorescence single nucleic acid	0.21–1.05 × 10^5^ pfu/mL	0.2 pfu/mL	Yes	[101]

**Table 4:** Comparison of biosensor platforms for Zika virus detection.

Biosensor Type	Virus/Sample	Recognition Element	Dynamic Range	Detection Limit	Portability	Reference
Electrochemical	Zika	Immobilized monoclonal antibody	500 ng/mL	0.45 nM	Yes	[104]
Electrochemical	Zika	Specific envelop protein antibody	10 pM–1 nM	<10 pM	Yes	[105]
Optical	Zika	Envelope protein-coding region	5–500 pfu	5 pfu	Yes	[106]

**Table 5:** Comparison of biosensor platforms for Norovirus detection.

Biosensor Type	Virus/Sample	Recognition Element	Dynamic Range	Detection Limit	Portability	Reference
Optical	Norovirus	Anti-norovirus monoclonal antibody	0.01–100 ng/mL	0.01 ng/mL	Yes	[110]
Electrochemical	Norovirus (DNA)	DNA	1 pM–10 nM	8.8 pM	No	[111]
Optical	Norovirus	Norovirus-specific aptamer	20–1000 viruses/mL	30 viruses/mL	Yes	[112]

**Table 6:** Comparison of biosensor platforms for Influenza virus detection.

Biosensor Type	Virus/Sample	Recognition Element	Dynamic Range	Detection Limit	Portability	Reference
Electrochemical	Influenza A virus subtype H9N2	Anti-matrix protein 2 antibody and Fetuin A	8–128 HAU	8 HAU	Yes	[115]
Electrochemical	Influenza A virus	Immobilized DNA	1–10 nM	0.5 nM	No	[116]
Optical	H5N1 influenza virus	Anti-recombinant hemagglutinin protein of H5N1 aptamer	2–200 ng/mL	3.5 ng/mL	Yes	[117]
Optical	Influenza A virus	Not available	10–50 fM	Not available	No	[118]
Optical	Avian influenza virus H5N1	Aptamer specific against H5N1	0.128–1.28 HAU	0.128 HAU	Yes	[119]

**Table 7:** Comparison of biosensor platforms for Dengue virus detection.

Biosensor Type	Virus/Sample	Recognition Element	Dynamic Range	Detection Limit	Portability	Reference
Electrochemical	Dengue serotype 2	Specific peptide nucleic acid	1–100 fM	10 fM	No	[123]
Electrochemical	Dengue serotype 2 (NS1 protein)	Specific peptide	0.025–3.5 μg/mL	0.025 μg/mL	No	[124]
Electrochemical	Dengue virus (DNA)	Specific DNA probe	1 × 10^−6^–1 × 10^−12^ M	2.7 × 10^−12^ M	No	[125]
Optical	Dengue virus	Immobilized antigen	Not available	Not available	No	[126]

**Table 8:** Comparison of biosensor platforms for other virus detection.

Biosensor Type	Virus/Sample	Recognition Element	Dynamic Range	Detection Limit	Portability	Reference
Optical	Human adenovirus (DNA)	Primer DNA	1 × 10^1^–10^6^ cells/100 μL	10^1^ copies/reaction	Yes	[130]
Optical	Enterovirus 71	Major capsid protein VP1	8.1 × 10^5^–1.3 × 10^7^ vp/mL	67 vp/mL	Yes	[131]
Optical	Epstein–Barr virus	Oligonucleotide antigen	Not available	Not available	Yes	[132]
Optical	Apple stem pitting virus	Aptamer	1.0–1.0 × 10^−2^ μg/mL	10 ng/mL	Yes	[133]
Optical	Human papilloma virus 16 E7	Anti-human papilloma virus 16 E7 protein	0.021–15 ng/mL	2.87 ng/mL	Yes	[134]
Electrochemical	Human rhinovirus serotype 2	Molecularly imprinted polymer	4 μg/mL–3 mg/mL	Not available	Yes	[135]
Optical	Japanese encephalitis virus	Surface imprinted polymer	1.2–960 pmol/mL	240 pmol/mL	No	[136]
